# An overview of coarse particle beneficiation of lithium ores

**DOI:** 10.1038/s41598-025-14059-z

**Published:** 2025-08-08

**Authors:** Philipa A. Opoku, Bogale Tadesse, Boris Albijanic, Aleksandar N. Nikoloski

**Affiliations:** 1 Western Australian School of Mines, Curtin University, Egan St, Kalgoorlie, WA 6430 Australia; 2 Harry Butler Institute (Centre for Water Energy and Waste), Engineering and Energy, Murdoch University, Perth, Australia

**Keywords:** Coarse particles, Dense media separation, Flotation, Magnetic separation, Ore sorting, Lithium ores, Spodumene., Surface chemistry, Chemical engineering

## Abstract

The increasing demand for lithium-ion batteries particularly for electric vehicles underscores the importance of improving the sustainability of lithium mining operations. The depletion of high-grade lithium ore deposits has necessitated the upgrading of medium to low-grade ores for lithium extraction. Spodumene is the most commercially exploited lithium-bearing mineral found in pegmatites due to its high lithium content. Ore sorting can be used for early rejection of up to 60% of gangue minerals prior to preconcentration. Dense media separation is a viable spodumene beneficiation method. However, as case studies have shown, flotation may still be required to process middlings and the undersized fraction, which falls outside the particle size range effective for dense media separation. Magnetic separation can be conducted during or after flotation to remove iron impurities in lithium concentrates. While fine particle flotation has historically achieved high recovery rates, their economic feasibility is increasingly questioned due to intensive comminution requirements. Coarse particle flotation in mechanical flotation cells for instance is inefficient due to turbulence-induced detachment of coarse particles. Coarse particle beneficiation using fluidized bed flotation cells can offer advantages such as reduced grind size and environmental footprint. Despite proven energy savings and recovery efficiencies in other mineral sectors, their application in lithium mining operations remains limited to pilot scale. Also, research in this area is underexplored. This review addresses this gap by evaluating the feasibility, potential benefits and challenges of integrating ore sorting, dense media separation, magnetic separation and fluidized bed flotation with the HydroFloat, NovaCell and Reflux cells into lithium ore beneficiation flowsheets. Key challenges identified include high water consumption and the inadvertent entrainment of fine particles requiring desliming steps. Furthermore, this review acknowledges the challenges in spodumene beneficiation due to the structural similarities among silicate minerals and highlights relevant pretreatment methods to improve selectivity, recovery and grade.

## Introduction

**Fig. 1:**
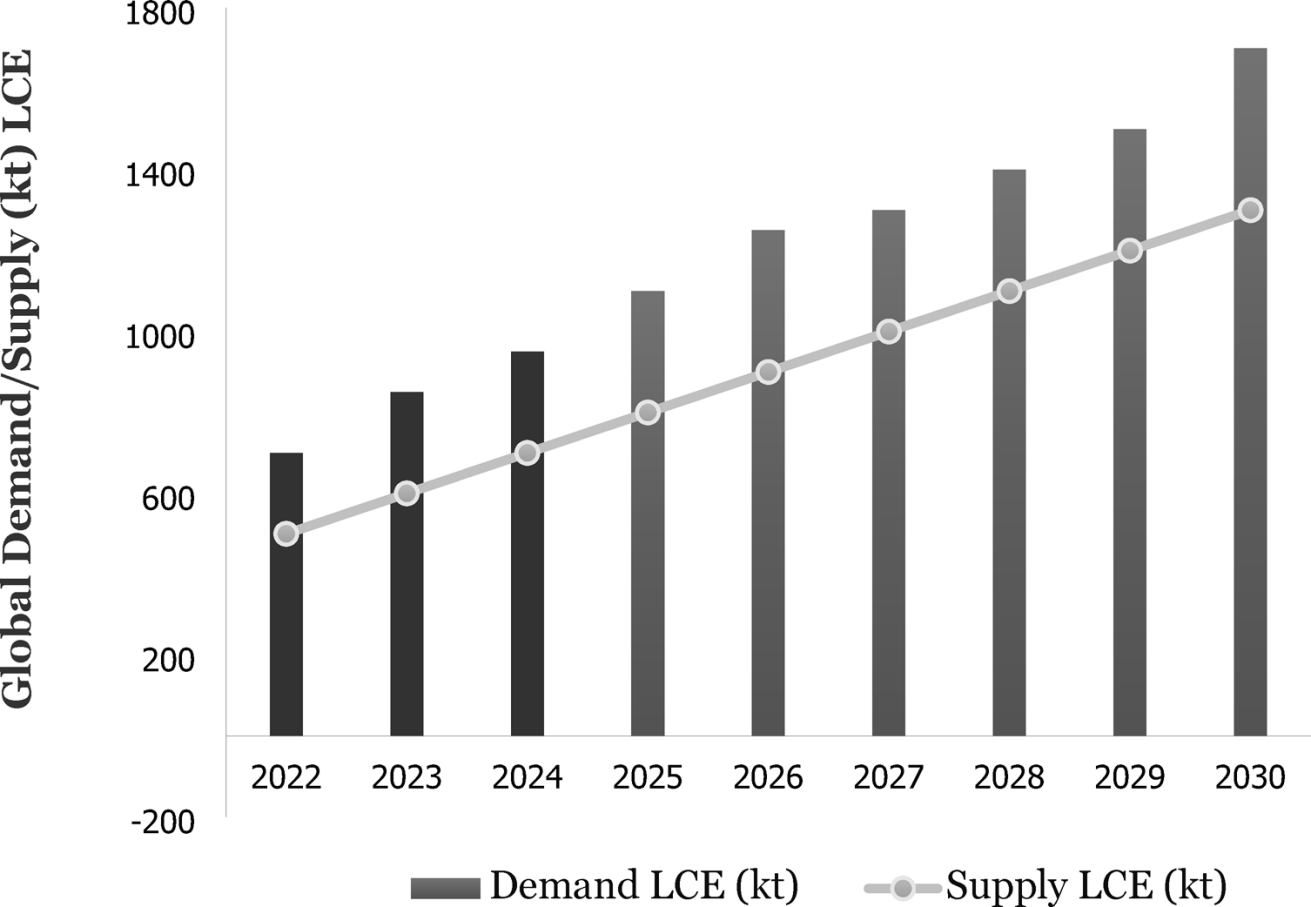
Global lithium supply and demand lithium carbonate equivalent (LCE)^2^.

**Fig. 2:**
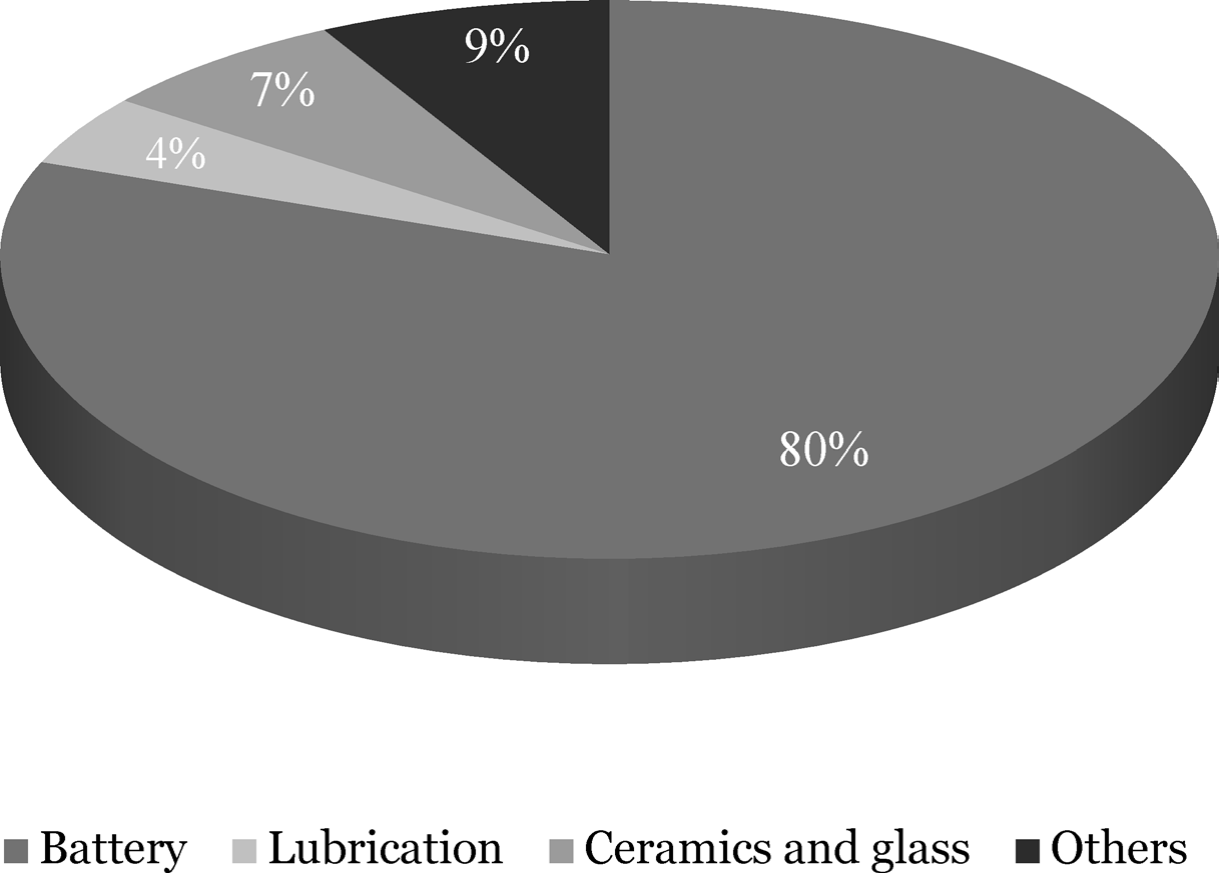
Major applications of lithium in 2022^4^.

Globally critical minerals such as lithium are in high demand. Lithium is the lightest alkali metal on the periodic table with an atomic weight of 6.939 g/mol and a density of 0.53 g/cm^3^. Its unique physical and chemical properties such as high conductivity, chemical reactivity and specific heat make it a valuable component in the production of batteries, lubricants, ceramics and glass^1^. Driven by its critical role in battery production, global lithium demand has been steadily increasing at approximately 20% per year^2^ and is anticipated to grow significantly in the coming years as shown in (Fig. 1). The annual global lithium demand was projected to surge by a factor of 27–37 from 2012 to 2050^3^. In 2022, the battery sector accounted for about 80% of the global lithium consumption (Fig. 2).

Key sources of lithium minerals include brines (60%), pegmatites (25%) and sedimentary rocks^5^. Lithium-bearing minerals hosted in pegmatites include spodumene, lepidolite, petalite, amblygonite, zinnwaldite, triphylite and eucryptite^6^.

Spodumene (LiAlSi_2_O_6_) is the primary commercial source of lithium found in pegmatite deposits due to its high theoretical lithium content of about 8.0–8.2% Li_2_O^7^. The beneficiation of spodumene is challenging due to its close association with gangue minerals such as feldspar (4 K[AlSi_₃_O_₈_]), quartz (SiO_2_) and muscovite (K_2_Al_4_Al_2_Si_6_O_20_) which share similar mineralogical characteristics and surface properties^8^. Dense media separation (DMS) and froth flotation are the main techniques used in spodumene beneficiation^8,9^. The effectiveness of these techniques hinges on comminution-mediated liberation between valuable and associated gangue minerals. However, lithium mining faces sustainability challenges, as comminution accounts for approximately 75% of the sector’s energy consumption, representing 2–3% of global electricity use^10^. To mitigate the energy-intensive costs associated with comminution of lithium ores, it is imperative to deploy ore-specific beneficiation technologies effective at the coarse particle level.

Coarse particle beneficiation presents its own set of challenges. For instance, the flotation of coarse particles is hindered by poor liberation and reduced buoyancy within conventional flotation cells (CFCs). Numerous studies have highlighted the difficulties in coarse particle flotation^11,12^. However, studies by Awatey et al.^13^, Dankwah et al.^14^ and recent reviews by Kromah et al.^15^ and Janisha Anzoom et al.^16^ have shown that the limitations of CFCs in the flotation of sulphide minerals can be addressed by utilizing one of the three main Fluidized bed flotation cells (FBFCs): HydroFloat™ (HF), NovaCell™ (NC), and Reflux™ (RC). However, there is a lack of literature on the application of FBFCs in lithium operations.

This paper provides an overview of the coarse beneficiation methods applicable to lithium ores: (i) gravity separation (ii) coarse particle flotation, (iii) ore sorting and (iv) magnetic separation. The advantages and limitations of each separation method are discussed. In this review, particle sizes are defined in the range; coarse (+ 150 μm–2 mm), fine particles (+ 38–150 μm) and ultrafine (0–38 μm). While acknowledging the relevance and paucity of literature on other lithium bearing minerals, this review primarily focuses on applications related to spodumene beneficiation.

### Ore sample characterization

**Fig. 3:**
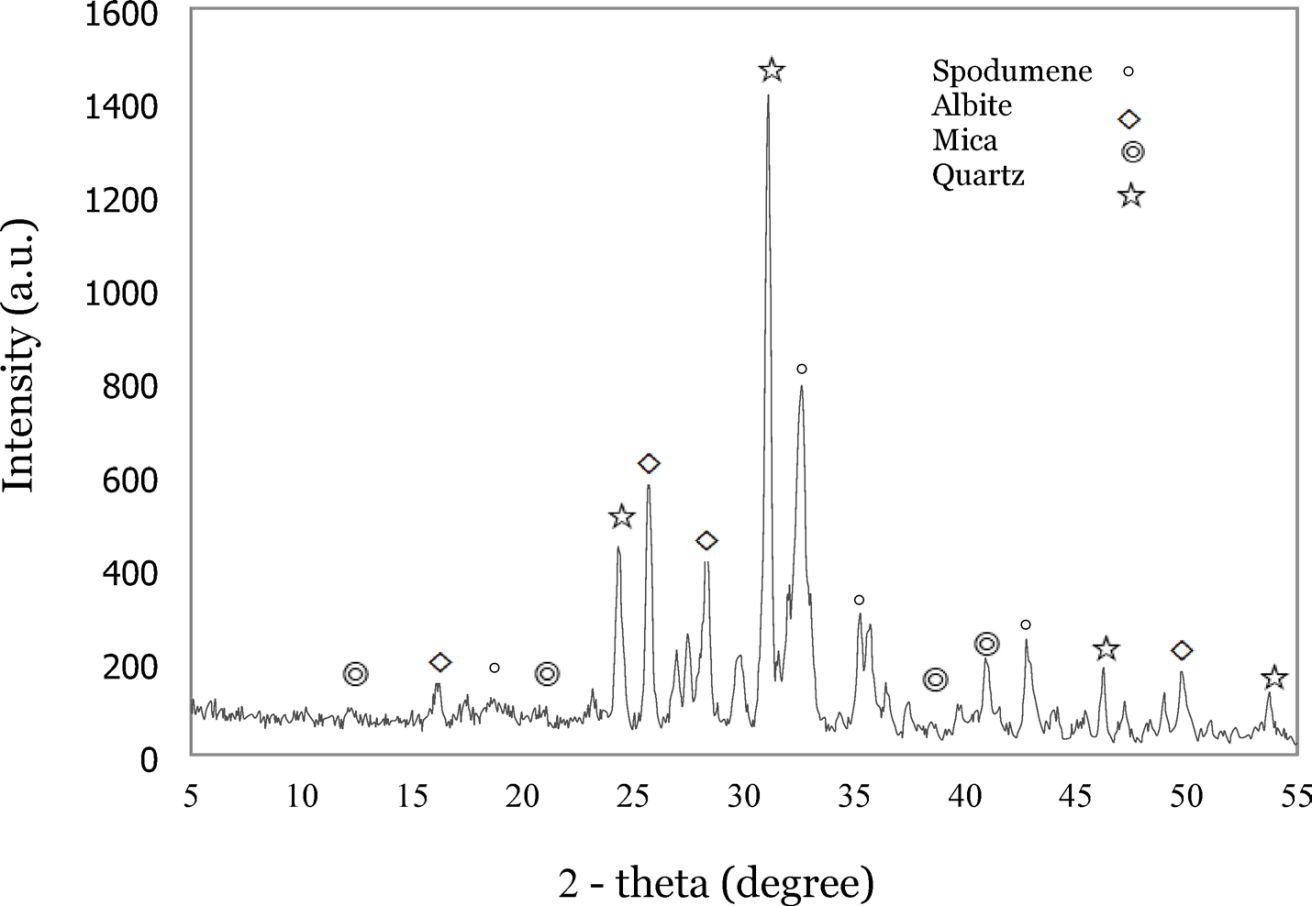
XRD pattern of pegmatite ore. (Courtesy of authors).

The pegmatite ore sample was sourced from a mine in Western Australia (WA). After crushing, the samples were ground and sieved to -48 μm. Representative subsamples were taken for mineralogical analysis. Quantitative X-ray diffraction (XRD) analysis of the ore sample was conducted using XRD Analyser (Olympus BTX II). The relative abundance of minerals present was quantified using Rietveld analysis, with a good fit between the observed and calculated pattern data (Rwp = 9.99%). XRD identified spodumene (9 wt%) as the lithium-bearing mineral. The major gangue minerals present in the samples were quartz, feldspar and mica as summarized in (Table 1). The XRD spectra is shown in Fig. 3.

**Table 1:** Major minerals present in pegmatite sample sourced from WA (wt%). (Courtesy of authors).

Mineral	Spodumene	Feldspar	Quartz	Mica	Amphibole	Pyroxene
Weight (%)	9	45	24	10	6	6

## Spodumene beneficiation

The initial run-of-mine (ROM) ores supplied to processing plant circuits typically contain coarse particles, ranging in size 1–100 mm. The techniques in spodumene beneficiation are highly dependent on ore mineralogy^8,17^ as well as the differences in the physical and chemical properties of associated minerals including specific gravity (SG), liberation and surface wettability^18,19^. Gravity separation proved efficient in preconcentrating high-grade lithium ores and rejecting coarse tantalite and quartz minerals^20^. Froth flotation is typically utilized for processing finer particle-sized ores, as the density variations can be too minimal for gravity separation. Magnetic separation can be utilized during or after flotation to remove iron-bearing gangue minerals such as magnetite, ilmenite, hematite and biotite to meet product quality specifications. For instance, battery-grade concentrates generally aim for lithium oxide (Li_2_O) levels above 6.0% and iron oxide (Fe_2_O_3_) below 1.0%^21^. Sensor based sorting offers advantages in spodumene processing through early waste reduction and efficient material routing into processing lines^22^. In some scenarios, an integrated approach, which combines some or all the key beneficiation methods may be necessary^23^.

It is well known that the efficient separation of minerals is significantly influenced by particle size. For instance, the efficiency of froth flotation declines as particle size increases due to insufficient liberation and buoyancy constraints within CFCs^24^. Other methods such as ore sorting, magnetic and gravity separation become less effective as particle size decreases.

### Gravity separation

Gravity separation, next to hand picking, is the oldest efficient mineral preconcentration method applied at coarser sizes up to 75 mm^25^. This method relies on the differential movement of particles within a fluid medium under gravitational or centrifugal forces^26^. Common conventional gravity separation (CGS) equipment includes shaking tables, jigs and spirals. Bale and May^18^ reported the use of spirals and shaking tables at the Greenbushes, WA to produce a spodumene concentrate free of cassiterite (SnO_2_) and tantalite (Ta_2_O_5_, Fe_2_O_3_). Spiral circuits were employed in the treatment of Bernic Lake pegmatite to remove and concentrate tantalum minerals as reported by Ferguson et al.^27^. However, CGS methods typically struggle to retain fine particles (−0.075 mm) due to insufficient residence time in the fluid^28^. The low settling velocities of fine particles in the fluid necessitate a significantly reduced processing rate. Consequently, in the size range of 0.01–0.1 mm, it has been essential to introduce centrifugal forces, either by developing a rotating suspension using a tangential feed entry or by mechanically rotating the system^29^. Enhanced gravity separators (EGS), which incorporate centrifugal forces alongside gravity, have been developed to improve the separation efficiency of fine particles above 0.001 mm. Key examples of EGS equipment include the Kelsey Jig, Knelson concentrator, Multi gravity separator (Mozley C900 separators) and the Falcon concentrator. EGS technologies increase the settling rate of fine particles by creating a sharper density contrast between heavy and light minerals^30,31^.

The efficiency of gravity separation is influenced by the concentration criterion (CC)^25^. As the SG of the separation medium increases, the CC increases leading to high separation efficiency. Gravity separation is generally effective when CC is greater than 2.5^23^. In water, the CC for separating spodumene from other silicate minerals ranges from 1.18 for muscovite to 1.38 for K-feldspar which indicates challenges in achieving effective separation even under optimal liberation conditions. However, an increase in the SG of the liquid can enhance the CC for all silicate gangue minerals. Gibson et al.^9^ found that when a heavy liquid with SG of 2.5 was used as medium, the CC values between spodumene and various gangue minerals ranged from 2.00 to 11.8. This showed that spodumene could be effectively separated from feldspar (CC = 11.8), albite (CC = 5.20) and quartz (CC = 4.26). However, there were still challenges with effective separation from muscovite as the CC value was still below 2.5.

### Spodumene concentration with DMS

DMS, also known as HLS or the sink and float method, is a widely adopted gravity concentration technique in lithium processing plants. This method is frequently employed for the early rejection of gangue in the coarse particle size range of 0.5–75 mm. DMS utilizes a heavy liquid referred to as dense media, which has SG that falls between that of spodumene and the associated gangue^8^. The average SG of spodumene is 3.15, slightly higher than that of quartz (2.65), feldspar (2.60) and muscovite (2.8). This density contrast enhances separation efficiency in DMS, which relies on both particle size and SG differences for optimal performance^9^. Munson and Clarke^32^ identified three primary challenges associated with DMS in the beneficiation of spodumene: (i) the small differences in SG between spodumene and various silicate gangue minerals make separation difficult, (ii) the transformation of spodumene into micas through metamorphic decay and into clays with lower SG due to weathering impedes separation efficiency and (iii) the tendency of spodumene to fracture into acicular particles affects recovery. Despite these challenges, DMS has proven its viability as a pre-concentration method for spodumene.

Historically, the early application of DMS for spodumene pre-concentration was reported by Munson and Clarke^32^. This marked the deployment of the first DMS plant for processing spodumene-bearing pegmatites at the Edison Mine in the USA in 1949. The process utilized a dense media, a mixture of ferrosilicon and magnetite, with a SG of 2.70 to achieve a spodumene concentrate assaying 5.36% Li_₂_O and recovery of 47%. Subsequently, Redeker^33^ reported the implementation of DMS at a spodumene pegmatite deposit in North Carolina, USA, achieving a concentrate grade of 3.5% Li_₂_O, 50–60% recovery at feed size − 10 mm + 212 μm and a head grade of 1.3% Li_₂_O. However, the DMS operations faced critical challenges with reduced lithium recovery, leading to their decommissioning and replacement with flotation units in the early 1970s. The beneficiation flowsheet of the Bernic Lake Lithium Operation in Canada employed DMS to produce a spodumene concentrate free from feldspar and tantalum^34^. Amarante et al.^35^ reported the application of HLS at a spodumene processing plant in Northern Portugal, where bromoform was used as the heavy media to process feed size (0.075–0.3 mm), effectively separating spodumene from feldspar, quartz and muscovite. HLS successfully produced a sink product assaying 5% Li_₂_O with 61% recovery. Gibson et al.^9^ conducted DMS tests on + 840 μm pegmatite feed to produce a concentrate with a grade of 6.11% Li_₂_O and 50% Li recovery. However, the recovery of lithium in the spodumene concentrate was 6.7% lower for DMS compared to initial HLS tests, with recoveries of 49% and 56% respectively. This difference was attributed to process scale-up. All products were subjected to flotation and achieved final concentrate assaying 6.4% Li_₂_O and 80% recovery.

Sagzhanov et al.^36^ investigated the beneficiation of low-grade spodumene ore from Eastern Kazakhstan using DMS. The primary gangue minerals in the spodumene ore were feldspar, quartz and mica. For the coarse particle fraction of -1000 + 850 μm, and head grade 0.6% Li₂O, DMS produced a concentrate grade of 5.7% Li_₂_O at 90% recovery. Ahmed and Zaghib^20^ recently reviewed the gravity concentration unit for processing spodumene-pegmatite ore in Amareshwar, India. The units included Jigs, Shaking table and Falcon concentrators. Their findings shows that HLS yielded the highest separation efficiency, with increase in Li content from 1.10% to (4.0–4.4% Li_₂_O) in the concentrate. The performance was followed by Jigs which achieved 3.42% Li₂O. The Falcon concentrator and Shaking table yielded poor recoveries which was attributed to their relatively low CC. According to Kundu et al.^8^ the superior performance of HLS on coarser particles further underscores its potential as a preconcentration method for lithium bearing ores.

### Current industrial projects

In Australia, the major producers and exporters of spodumene concentrate from pegmatite deposits are the Greenbushes, Mt Cattlin and Mt Bald in WA^17^.

#### The Mount Cattlin spodumene project, WA

**Fig. 4:**
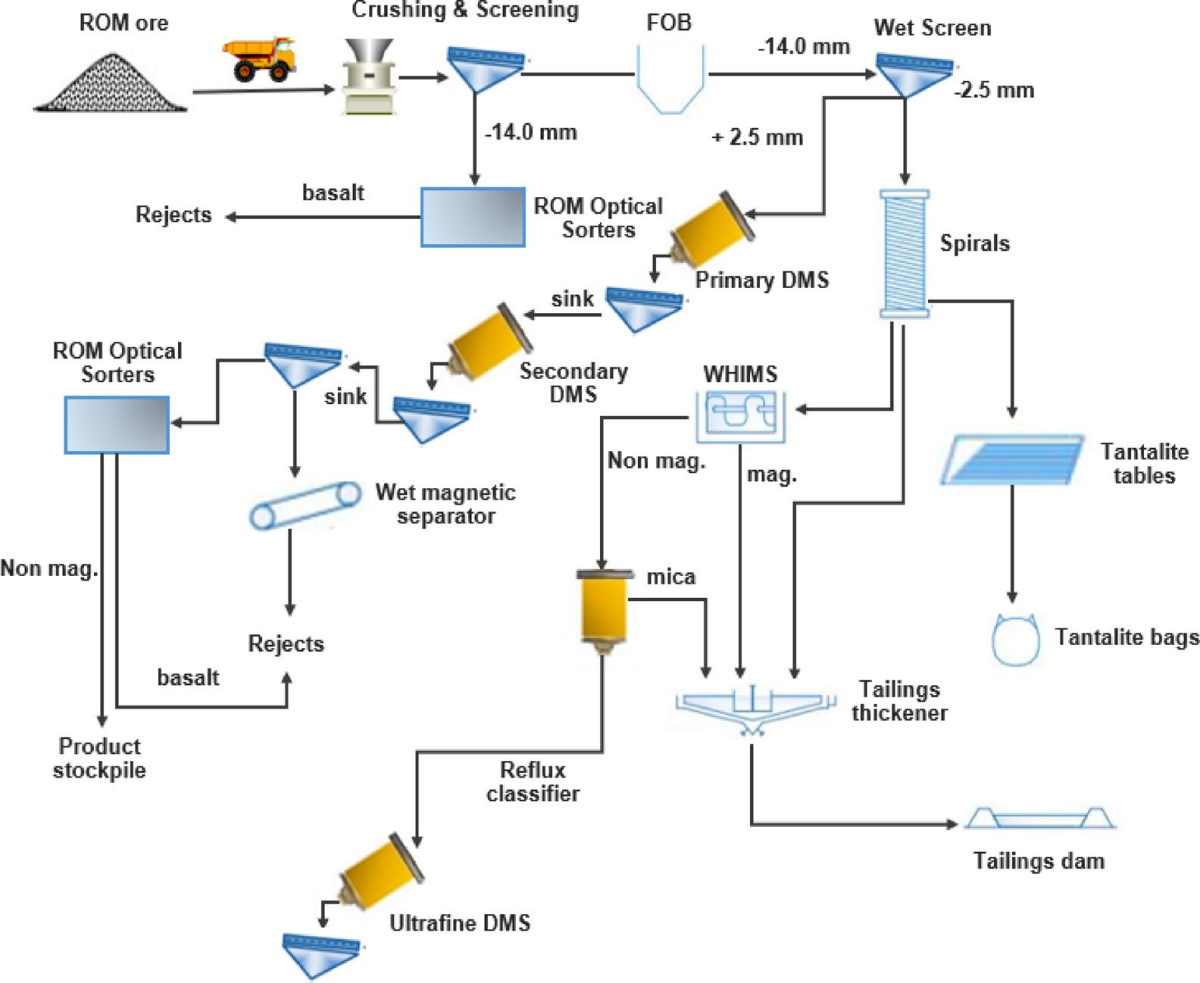
Modified Mt Cattlin process flowsheet^37^.

The Mount Cattlin Spodumene Project owned by Galaxy Resources, is located 2 km north of Ravensthorpe. The site produces spodumene concentrate and tantalite as by-product at a production capacity of about 180 kTPA of Li_₂_O concentrate^6^. Figure 4 presents the Mt Cattlin flowsheet implemented since March 2021^37^. The ROM ore is stockpiled and fed via a grizzly feeder into a primary jaw crusher. Oversize material (+ 125 mm) is crushed and conveyed to a secondary cone crusher. The crushed product is screened, with the + 50 mm fraction returned to secondary crushing and the − 50 mm fraction sent for further classification. When processing contaminated ore, material is routed through two triple-deck sizing screens (40 mm, 22 mm and 14 mm apertures) to generate feed for optical sorting.

The + 40 mm fraction is treated by optical sorters to reject basalt while the − 14 mm fraction reports directly to the Fine Ore Bin (FOB). Material from the FOB is wet screened at 2.5 mm. The + 2.5 mm oversize is classified into (+ 2.5–6 mm and + 6–14 mm) size fractions and fed to a 2-stage DMS circuit which uses ferrosilicon media. The sink (spodumene concentrate) is sent to the Product Quality Upgrade (PQU) circuit for basalt removal to improve the grade.

The − 2.5 mm undersize is further classified via Derrick screens into coarse (+ 800 μm-2.5 mm) and fine (-800 μm) fractions. The + 800 μm fraction is sent to coarse spiral concentrators, whiles − 800 μm is deslimed in cyclones then pumped into fine spiral concentrators. The concentrate from both fine and coarse spirals merge on a single Wilfley shaker table for tantalite removal. The waste product from the fine spirals is thickened and sent to tailings whiles that of the coarse spirals is sent to the ultrafine circuit for further Li_₂_O recovery. The coarse spirals waste is pumped to the Wet High Intensity Magnetic Separator (WHIMS), where magnetic material is discarded and non-magnetic material is directed to a reflux classifier for mica removal^37^.

#### The bald hill project, WA

**Fig. 5:**
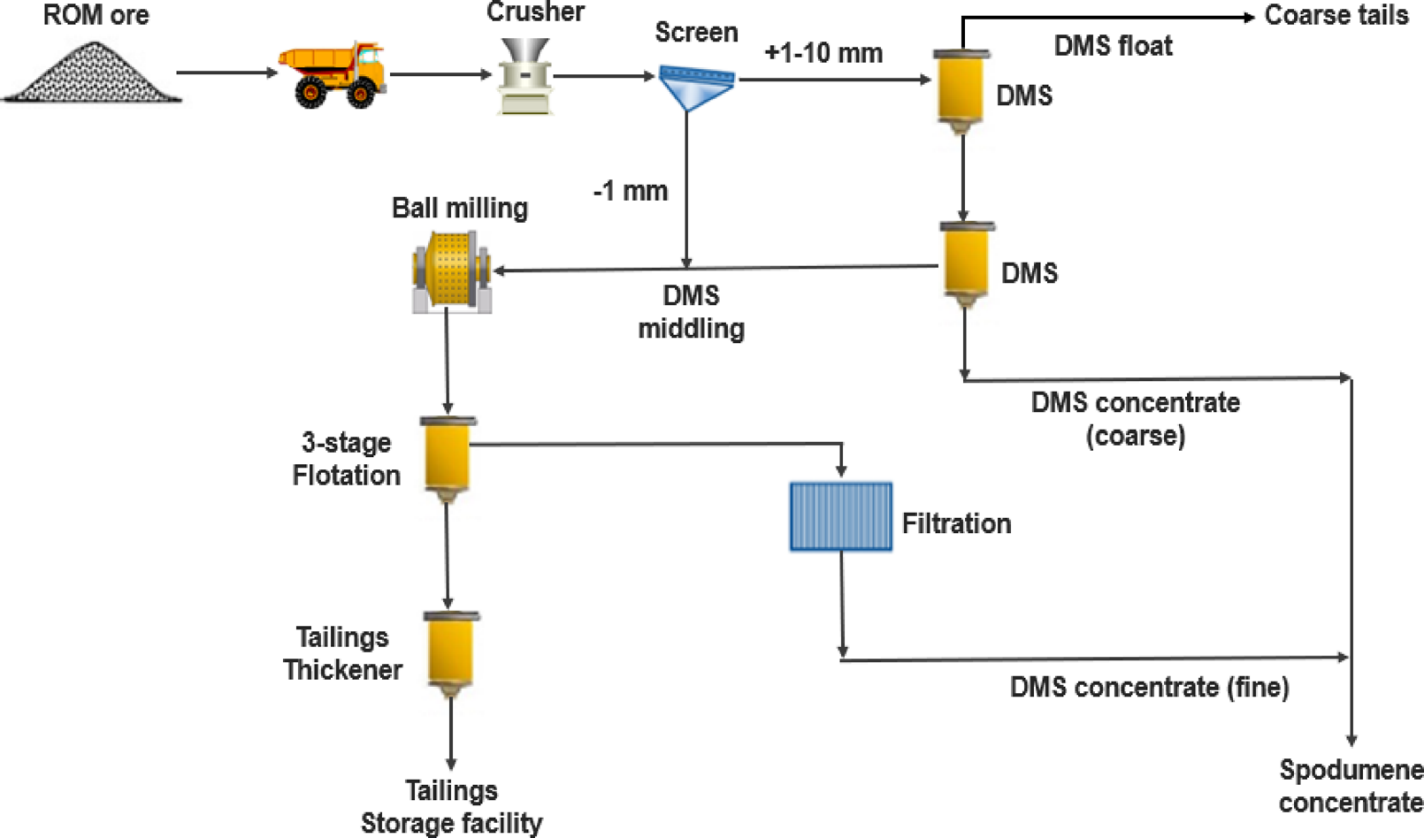
Schematic flowsheet for the Bald Hill plant^40^.

The Bald Hill processing plant utilizes DMS to produce about 1.2 Mtpa of spodumene concentrate from pegmatite ores^38^. As shown in Fig. 5, the ROM is crushed to a P_100_ of 10 mm, followed by wet screening to remove − 1 mm material. Tantalite is removed via rougher spiral circuits. The coarse fraction (+ 1–10 mm) is sent to a DMS cyclone with ferrosilicon as dense media. The DMS circuit includes reflux classifiers to remove mica as well as primary and secondary DMS cyclones to produce the spodumene concentrate. The first shipment of spodumene concentrate in early May 2018 had an average grade of 6.37% Li₂O, 0.5% Fe₂O₃ and − 0.4% mica. The second shipment of spodumene concentrate in late May 2018 similarly averaged + 6% Li_2_O and − 0.6% Fe₂O₃. The overall recovery and quality of the concentrate was not highly sensitive to feed grade which led to a reserve cut off from 0.39% Li₂O to 0.3% Li₂O. In 2019 the plant was shut down due to low recovery, low prices of products and no flotation units. Flotation units were installed and the DMS plant came on stream^39^.

#### The Grota do Cirilo lithium project, Brazil

**Fig. 6:**
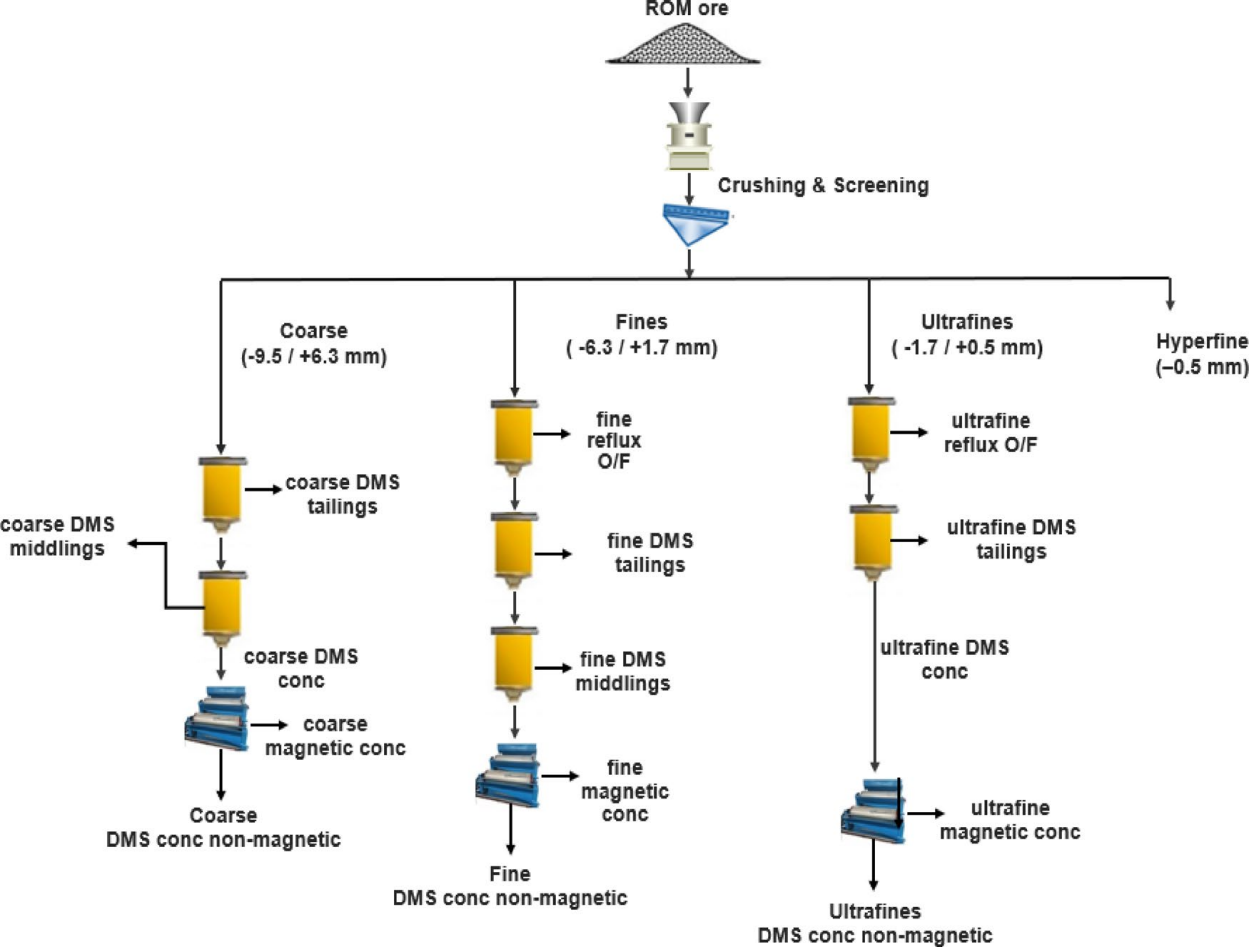
Simplified flowsheet of the Grota do Cirilo Lithium Project adapted from Delboni et al.^41^.

The Greentech plant incorporates a DMS circuit to concentrate coarse pegmatite particles (+ 5.5 mm)^41^. These pegmatites consist of microcline, quartz, spodumene, albite and muscovite. The spodumene content ranged from 28 to 30% in the dike structure. For the initial tests, the goal was to produce spodumene concentrate with grades of 6% Li_2_O and 1% Fe_2_O_3_. Four HLS tests, at four crush sizes (15.9 mm, 12.5 mm, 9.5 mm and 6.3 mm) were conducted. The tests were successful, and the 9.5 mm crush size was identified as optimal for further DMS test work due to its higher lithium recovery and minimal fines generation. Subsequently, each DMS sample was crushed to -9.5 mm and screened into four distinct size fractions: coarse (-9.5 mm + 6.3 mm), fines (-6.3 mm + 1.7 mm), ultrafine (-1.7 mm + 0.5 mm) and hyperfine (-0.5 mm). Subsequent magnetic separation stage was conducted to further refine the concentrate. Figure 6 illustrates a typical flowsheet for the plant.

Some pilot studies on DMS for spodumene beneficiation are summarized in Table 2.

**Table 2:** DMS applications in spodumene beneficiation.

Dense media	Particle size (mm)	Concentrate assay recovery (%), Grade (%Li_2_O)	References
Media with SG (2.6- 3.0)	+ 0.5 − 9.51	45, 6.4	^42^
Aqueous suspension of ferro-silicon	+ 1–6	50, 6.8	^43^
Ferrosilicon and water	+ 0.5−9.5	90, 5.7	^44^
Ferrosilicon and magnetite	+ 0.840	50, 6.11	^9^
Water	−6.4 + 3.3 −3.3 + 1.0	37, 5.7 53, 6.4	^45^
Ferrosilicon and water	0.850	6125 mg/kg	^46^
Ferrosilicon	+ 0.85−1	90	^36^

It is important to note that DMS alone may not be effective for lithium ore beneficiation^47^, as evidenced in Bald Hill project. In such cases there has been the need to integrate flotation techniques into the beneficiation flowsheet^27^.

## Flotation

Froth flotation involving mechanically agitated cells has proven effective in concentrating spodumene ores with lower-than-optimal grades, particularly within the fine particle size range of + 38–150 μm^48–50^. For spodumene flotation, alternative technologies including mechanical flotation cells, coarse particle flotation cells, column flotation cells and Staged Flotation Reactors (SFR) can be utilized independently or in conjunction to enhance recovery, selectivity and process efficiency^47^. The primary methods for spodumene flotation include: (i) direct flotation of spodumene using anionic collectors and (ii) reverse flotation of gangue minerals such as feldspar, quartz and mica using cationic collectors^51,52^. Tian et al.^53^ developed a scheme to improve spodumene flotation by recycling mica and feldspar from lithium tailings by reverse flotation. They recommended the flowsheet which involved; primary grinding - desliming - coarse mica flotation - spodumene roughing - rough concentrate regrinding and reconcentrating, which improved the grade and recovery of Li_₂_O in spodumene concentrates to 6.02% and 87.34%, respectively from initial assay of tailings grade 1.43% L_i2_O.

The flotation of spodumene in real ores is particularly challenging due to the similarity in flotation behaviour with associated gangue minerals, particularly feldspar. This similarity along with their close points of zero charge; spodumene (2.0)^54^, feldspar (1.7), quartz (2.0)^50^ hinders the selectivity achievable with current collectors.

One of the primary challenges in spodumene flotation is the use of fatty acid collectors, which, though have collecting ability, exhibit limited selectivity^55^. Common commercial collectors for spodumene include tall oil fatty acids (TOFAs) and synthetic fatty acids^56^. Oleic acid in its sodium salt form (NaOL) is frequently used in research on spodumene flotation. However, the inherent foaming properties of fatty acids can further hinder flotation selectivity by entrainment of fine gangue particles^21^. In some instances, increasing collector dosage, choosing collectors with higher contact angles and utilizing mixed collectors have been shown to enhance spodumene hydrophobicity^19^. Studies on the effects of mixed collectors to improve spodumene flotation is summarized in Table 3.

**Table 3:** Effect of mixed collectors on spodumene flotation.

Single collector	Mixed collector	Recovery (%)	References
	NaOL/dodecyl ammonium chloride (DTAC) 9:1	85	^49^
	NaOL/DTAC 9:1	89	^57^
	YOA (a mixture of 90% oleic acid and 10% DDA)	89	^50^
NaOL	NaOL/tributyl tetradecyl phosphonium chloride (TTPC) 5:1	72 55	^58^
	NaOL/DDA 6:1 OHA/DDA 6:1	86 < 30	^59^
	NaOL/dodecyl succinimide (DS) 5:1	90	^3^
Hexyloxypropylamine (HPA) N-Dodecyliminodiacetic acid (DIDA)	HPA/DIDA 1:3	80 0.1 93	^60^
Decyloxy-propyl-amine (DPA) α-bromododecanoic acid (α-BDDA)	DDA/α-BDDA 1:19	47 75 86	^51^
	DDA/NaOL 1:6	80	^61^
	NaOL/cetyl trimethylammonium bromide (CTAB) 7:1	73	^62^
NaOL	NaOL/N-lauryl-1, 3-propylene 5:1	80 51	^63^
	Fe-octyl hydroxamic acid (OHA) (iron complexes of octyl hydroxamic acid	84	^64^
	NaOL/Sodium dodecyl sulfonate (SDS) 1:1	90	^65^

### Effect of pretreatment conditions on spodumene flotation

Reviews by Xie et al.^51^, Salakjani et al.^66^, and Cui et al.^67^ emphasized that selective pretreatment techniques such as grinding in alkali solution, surface dissolution and isomorphous substitutions can significantly improve spodumene flotation. The different spodumene crystal planes exhibit varied affinities for collectors^68^. The anisotropic properties of spodumene demonstrate varying degrees of hydrophobicity on its crystalline planes {110} and {001}. Different grinding media (ball, rod, autogenous) and conditions (wet, dry) can influence the cleavage and fracture of crystal structures. Notably, the {110} cleavage plane of spodumene exhibits a high contact angle, indicating the preferential chemisorption of anionic collectors like sodium oleate onto surface aluminium (Al) sites^69^.

Rai et al.^70^ measured the contact angles on spodumene surfaces using the free sessile air bubble method. They found that the contact angle for the (110) surface plane was larger than that for the (001) surface with NaOL collector. Studies by Zhu et al.^71^ reported that wet-ground spodumene had smoother surfaces with increased exposure of {110} and {100} planes than dry-ground samples which showed higher exposure of {010} planes. Xu et al.^72^ also investigated the surface wettability differences across anisotropic crystal surfaces of spodumene using contact angle measurement, atomic force microscopy (AFM) and density functional theory (DFT) calculations. They ranked the hydrophilicity of the crystal planes in the order (010) > (100) > (110).

Some studies have shown that wet grinding in alkali media can significantly influence spodumene recovery^73,74^. Pretreatment with NaOH has been found to enhance adsorption of anionic collectors by increasing Al^2+^ surface sites through additional Al-O bond breakage. Yu et al.^75^ investigated the effects of grinding spodumene in Na_₂_CO_₃_, followed by stirring in NaOH. This treatment led to selective dissolution of spodumene surfaces, reduced slime interference and enhanced collector interaction. Ma et al.^74^ also observed that the flotation kinetics of spodumene followed a first-order model, with an enhanced flotation kinetic constant after alkali pretreatment with NaOH. Further analysis revealed a significant increase in Al active sites on the spodumene surface compared to feldspar after pretreatment. However, it is important to note that pretreatment with alkali may result in the generation of high-alkali wastewater which is difficult to treat. Hence, spodumene flotation under low-alkali conditions may be a viable strategy to mitigate this issue^67^.

Nazir et al.^76^ reported that calcination prior to flotation enhances spodumene brittleness, facilitates gangue rejection and improves lithium recovery and grade in finer fractions. However, despite these benefits, the high energy requirements for calcination may offset its benefits. Studies by Chu et al.^77^ show that spodumene particles pretreated by ultrasound within particle size range − 0.15 + 0.074 mm and − 0.074 + 0.0385 mm exhibit a stronger ability to adsorb NaOL compared to those treated by traditional mechanical agitation. Under traditional mechanical agitation, the amount of NaOL adsorbed on spodumene was 1.5 mg/g at a NaOL concentration of 200 mg/L. In contrast, ultrasonic pretreatment increased the collector adsorption to 2.1 mg/g. For fine particles below 0.0385 mm, the study found no significant difference in NaOL adsorption between the two pretreatment methods across five different collector concentrations. Although more collectors were adsorbed onto the fine particles surface (− 0.0385 mm), flotation recovery was lower compared to that of coarse particles (− 0.15 + 0.0385 mm). This disparity was attributed to the larger specific surface area of fine particles, which reduces collector density on the mineral surface.

The addition of metal ions plays a crucial role in spodumene flotation and understanding their interaction mechanisms is vital for optimizing recovery^68,78^. The use of metal ions for spodumene activation has become a common practice in flotation. Jie et al.^68^ investigated the effect of Fe³⁺ on the flotation of spodumene, albite and quartz. They found that flotation was challenging with NaOL alone. However, with the addition of Fe^3+^ selectively activated spodumene. Zeta potential measurements showed that Fe^2+^ reduced the negative charge on the mineral surfaces at pH < 8, increased NaOL adsorption and improved flotation recoveries. Liu et al.^48^ observed enhanced floatability of spodumene with Ca^2+^ at pH 12.5 and Mg^2+^ at pH 10.0, which they attributed to the formation of CaOH⁺ and MgOH^+^ complexes, as well as Ca(OH)_2_ and Mg(OH)_₂_ precipitation in strongly alkaline conditions. However, Xu et al. (2016) observed that in the absence of depressants, both spodumene and feldspar were activated by Ca^2+^, leading to a lack of selectivity in flotation. To achieve selectivity, Na₂CO₃ was required as a depressant as suggested by^56^. Xie et al.^51^ also reported that, Ca^2+^, Mg^2+^, Cu^2+^ and Fe^2+^ improved the selectivity of spodumene against feldspar and quartz, with Ca^2+^ and Mg^2+^ showing the highest selectivity. Meng et al.^78^ confirmed that Fe^2+^, Mg^2+^, and Ca²⁺ shows strong activation of spodumene at pH 6.5, 10.9, and 12.1, respectively, however, Mg^2+^ and Ca^2+^ shows superior activation compared to Fe^2+^.

Lin et al.^65^ studied the activation mechanism of Mg^2+^ with alkali treatment for spodumene and feldspar. They observed that after Mg^2+^ dissolution, the unsaturated O site on the mineral surface was the primary site for collector adsorption. These findings emphasize the importance of metal ions in enhancing spodumene flotation from silicate minerals and underscore the need for further studies on their activation mechanisms. Some pretreatment methods and their effect on spodumene flotation is presented in Table 4.

**Table 4:** Effect of pretreatment conditions on spodumene flotation recovery.

Effect of grinding media	Grinding media	Recovery (%)	References
	Zirconium balls Iron balls	60 50 80 60	79
	Zircon ball Iron ball	68 55 90 65	80
	Small nylon pot Small corundum pot Large corundum pot	91 90 78	81
	Rod milled products Ball milled products	80.7 40.3	82
	Corundum balls Agate balls	80 70	61
Effect of particle size	Particle size (µm)		
	38–45^a^ 45–75^b^ 19–38^c^ 0–19^d^	a > b > c > d	49
	−80 + 40^a^ −150 + 80^b^	a > b	54
	−75 + 45^a^ −45 + 31^b^ −31 + 19^c^ −19 + 0^d^	a > b > c > d	7
Effect of surface dissolution	Acid/Alkali		
	NaOH Na_2_CO_3_	NaOH showed an activation effect, but Na_2_CO_3_ depressed the flotation of spodumene	50
	NAOH HCl	Recovery NaOH treatment > no treatment > HCl treatment	73
	NaOH	Increase in recovery (1.11%) grade (6.99%)	74

## Coarse particle flotation (CPF)

CPF is increasingly recognized as an effective technique for the early rejection of coarse gangue particles above 2 mm. Research shows that optimizing the upper size limit in primary grinding mills can achieve energy savings of 40–50%^83^ and enhance the mill capacity by up to 25%^84^. CPF can be highly beneficial given the energy-intensive nature of fine grinding in lithium operations^2^.

### Limitations in coarse spodumene flotation

#### Effect of particle size

The critical grind size is a characteristic specific to an ore, below which total recovery is attainable by flotation and above which flotation recovery starts to decline^24^. While the lower and upper size limits of floatable particles may vary based on particle density, recovery tends to decline as particle size increases, regardless of mineral composition or flotation cell design^85^. Early studies by Gaudin et al.^11^ attributed the detachment of coarse particles from bubbles to turbulence created by high agitation in mechanical flotation cells. Sufficient turbulence is necessary for particle dispersion and bubble-particle collision. However, it also generates eddies in the system. When bubbles become trapped in the centre of these eddies, they rotate rapidly leading to coarse particle detachment^24^. As a result, a significant amount of valuable minerals in the coarse spodumene can be lost to tailings. Filippov et al.^54^ investigated the flotation performance of spodumene using NaOL as a collector across two particle size fractions + 80–150 μm and + 40–80 μm. Their findings show that the recovery of spodumene was significantly higher for the finer fraction (+ 40–80 μm). XRD analysis indicated substantial differences in the relative intensities of crystal planes (110), (010), (100) and (001) between these size fractions, with the + 40–80 μm fraction exhibiting an elevated number of the (110) plane, which facilitated enhanced NaOL adsorption. Further SEM analysis of randomly selected (110) surfaces showed that spodumene particles in the + 40–80 μm fraction had a reduced aspect ratio which facilitated better bubble-particle attachment relative to the + 80–150 μm fraction.

#### Effect of mineral liberation

The effectiveness of CPF is significantly influenced by the degree and distribution of particle liberation. Coarse particles often exhibit reduced surface liberation which limits the available surface area for reagent interaction, essential for sufficient hydrophobicity in flotation^17,83^. The spatial pattern of liberated zones is also critical in spodumene flotation. Localized liberation on specific areas of particle surface facilitates stronger and more stable attachment. Conversely, particles with more evenly dispersed liberation may demonstrate weaker attachment. Optimizing grinding methods to create targeted liberation patterns is crucial to enhancing the flotation of coarse spodumene particles^80^. Aylmore et al.^17^ conducted a mineralogical assessment on three pegmatite samples sourced from outcrops within Pilbara Minerals’ Pilgangoora Project to determine their theoretical grade and recovery. The study shows that primary gangue minerals including quartz, K-feldspar and albite could be efficiently rejected at a coarse grind size of -4 mm. They achieved theoretical recovery above 90% spodumene, with a Li upgrade from initial assays of (0.99–1.5 wt% Li) to (3.0–3.5 wt% Li). Their findings demonstrate that high recoveries are attainable even at coarser particle sizes given sufficient liberation.

#### Bubble-particle interaction

A key factor that limits the maximum floatable particle size is the interaction between bubbles and particles during flotation. The flotation process involves three main stages: (i) the attachment of particles to air bubbles in the pulp phase, (ii) the transfer of bubble-particle aggregates to the pulp-froth interface and (iii) the recovery of particles from the froth phase^16^. The probability of a particle being collected in the pulp phase is determined by the combined probabilities of bubble-particle collision, attachment and stability^86^. Research by Hassanzadeh et al.^87^ shows that collision efficiency increases with particle size. However, coarser bubbles tend to decrease the collision probability due to their smaller surface area-to-volume ratio. Coarse particles typically have shorter sliding times due to higher tangential velocities, which result in lower attachment probabilities compared to finer particles. Studies by Ralston et al.^88^ showed that induction time increases with particle size and decreased attachment probability for coarse particles in turbulent conditions. Increase in the ionic strength of process water can also compress the electric double layer between bubbles and particles^89^, reducing induction time and improving attachment efficiency in spodumene flotation. Furthermore, the stability of the bubble-particle aggregate dictates the maximum floatable size of the particles^90^ and depends on the balance between adhesive and detachment forces. As particle size and density increase, gravitational and centrifugal forces also increase, potentially causing detachment if these forces exceed the attachment force.

### Fluidized bed flotation technology - enhanced coarse particle flotation

In FBFCs, particle suspension is achieved through a combination of smooth and hindered settling conditions created by the interactions of suspended particles^91^. A comparison of different FBFC-s is given in Table 5. The key advantages of FBFCs over CFCs is the lack of turbulence and ability to efficiently separate coarse-sized particles, high buoyancy, high retention time, efficient bubble particle interactions and low operational cost^15^. Unlike mechanical flotation cells, FBFCs do not require high particle contact angles for flotation. This enables them to achieve superior recovery at the same contact angle. Research on FBFCs technologies have focused on sulfide and base metal ores to efficiently separate mineral particle sizes up to 2 mm^13,92^. However, commercial adoption of FBFCs in spodumene processing is limited, with few applications reported in pilot projects. Sahoo et al.^2^ in their review suggest that the HF and RC flotation cells, can be tried for beneficiating coarse liberated lithium-bearing minerals.

Despite the advantages, FBFCs have operational challenges. One of the main drawbacks is the dependency on water for fluidization, which affects operations particularly in arid regions. For example, the HF was designed with the assumption that coarse particles would naturally detach from the froth and a froth depth would not form. However, the lack of a robust froth layer constrains HF performance and necessitates pre-feed size classification to remove fine particles^93^. FBFCs require high solid feed content that must be carefully regulated to ensure effective bubble-liquid segregation. This segregation is essential for transferring particle-laden bubbles to the froth phase, while providing sufficient residence time to enhance process kinetics^15^. Several operational parameters including fluidized bed height, expansion, gas holdup and porosity, as well as superficial gas velocity, superficial water velocity, feed rate, feed size distribution, bubble size and reagent concentration are crucial in the effective functioning of FBFCs^16^. Minor adjustments to these key parameters can significantly affect overall flotation performance. Estimating energy flows and efficiency in fluidized bed system is critical for optimizing process design. During fluidization, the input energy can be divided into two components: one that induces turbulence in the liquid phase and the other lost due to friction between the liquid and solid phases^94,95^. Panneerselvam et al.^96^ reported that during fluidization, the energy transfer efficiency from the liquid phase to the solid phase ranged from 80 to 90% and the power consumption in a stirred tank contactor was approximately three times higher than that in a fluidized bed reactor under comparable operating conditions.

#### HydroFloat™

The HF separator is the first fluidized bed separator designed in the early 2000s. This flotation technology introduces a quiescent, aerated fluidized bed design specifically engineered to mitigate the turbulence associated with CFCs^97^. The HF has the advantage of operating with minimal froth which maximizes the recovery of coarse particles by minimizing the need for buoyancy to penetrate the froth layer^98^. This design feature also reduces detachment of marginally hydrophobic coarse particles (+ 0.15–2 mm) upon impact with the pulp-froth interface. Other features of the HF that offer advantages over mechanical cells in CPF include (i) the combined advantages of flotation and gravity separation, (ii) enhanced bubble-particle interaction in the fluidization zone, (iii) high residence time, (iv) bubble cluster formation which creates sufficient buoyancy to float coarse particles and (v) reduced turbulence in the cell prevents bubble-particle detachment^84,99^. Key challenge with the design of the HydroFloat, is the requirement of pre-flotation feed size classification to remove fine particles, to minimize entrainment. The application of the HF to spodumene flotation is very limited.

**Fig. 7:**
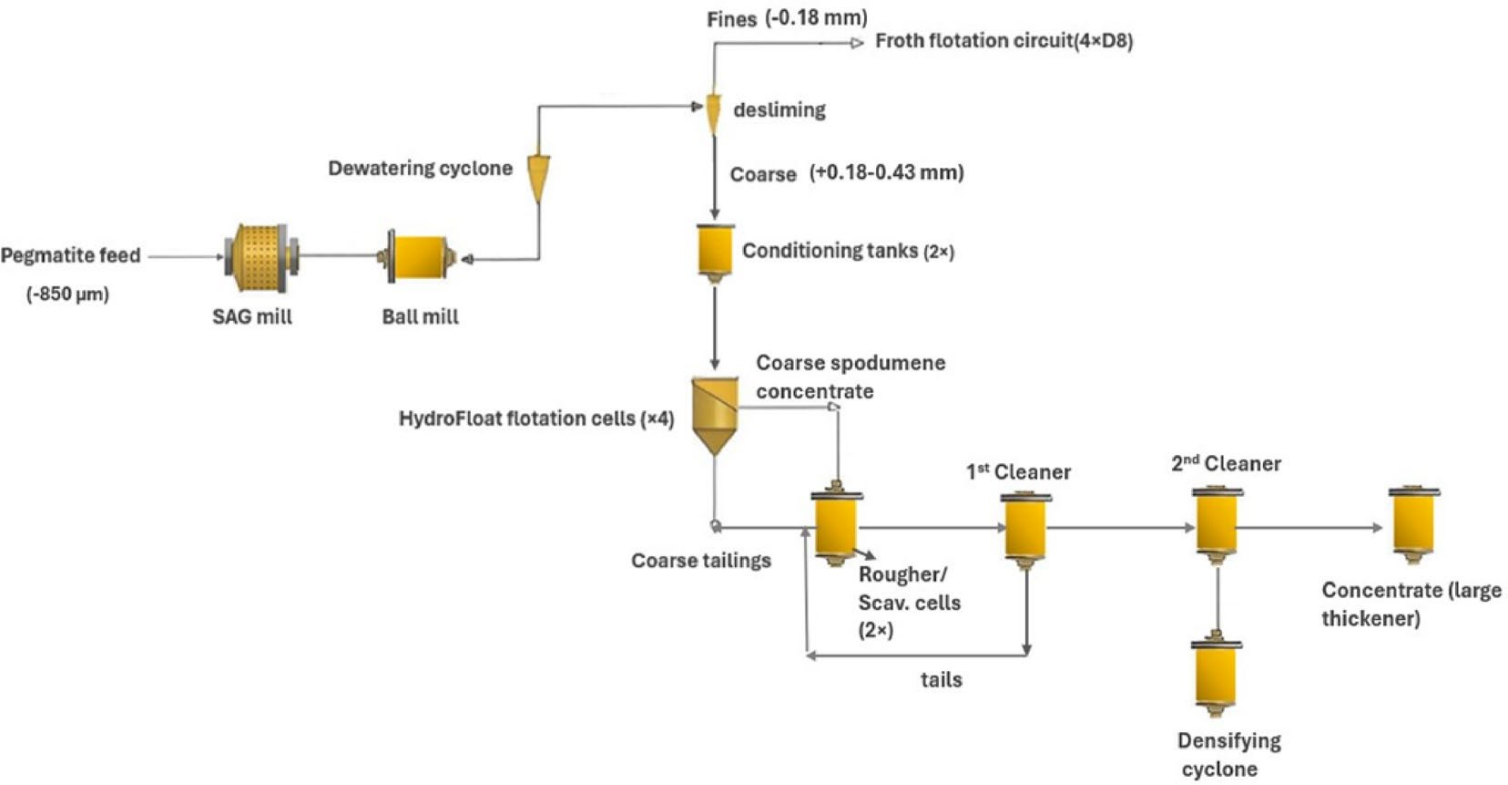
Modified pilot plant flowsheet with the HydroFloat circuit^100^.

According to a report by Nemaska Lithium Inc.^100^. , a feasibility study on the Whabouchi lithium mine and Shawinigan electrochemical plant was conducted using the HF separator to produce spodumene concentrate from pre-screened fines (< 850 μm). The feed was deslimed, conditioned and floated at coarse size (+ 0.18–0.43 mm). However, the separation was unsuccessful due to inefficient removal of fines from the screen oversize. This resulted in uneven reagent distribution, poor concentrate grade and recovery. Subsequent pilot plant tests replaced the HF with mechanical flotation cells and a cleaner cell. While the flotation columns achieved high grades up to 7.1% Li_2_O, recovery rates remained suboptimal. To address this, a hydraulic separator was introduced to remove fines from rougher flotation tailings, which produced an underflow with 1.16% Li_2_O. This underflow was re-floated using HF unit, but the resulting concentrate grade was only 3.55% Li_2_O, indicating limited upgrading efficiency of the HF unit. The study emphasized the importance of feed quality as the HF required high feed grade to achieve desired performance^101^. A section of the pilot plant flow sheet with the HydroFloat™ circuit is shown in Fig. 7.

#### NovaCell™

The NovaCell™ is an advanced flotation machine designed to capture particles across size range (-74 μm-2 mm), from the lower limit of flotation to an upper size that varies with the density of the mineral. Studies by Jameson and Emer^24^ showed that the NovaCell™ technology can achieve up to 40–50% in energy savings. However, it faces challenges with the accumulation of bubble-particle clusters that can become saturated beneath the froth phase. To address this, increasing the hydraulic lift or superficial water velocity can migrate these clusters into the froth layer^24^. The operation of the NC requires a cut-off velocity that ensures a stable fluidized bed while preventing coarse particles from being elutriated^15^. The design features a vertical column with a conical bottom section for fluidization and aeration, allowing fine hydrophobic particles to rise while coarse gangue settles. The mid-section facilitates the rise of bubble-particle aggregates, while the collection section provides two product streams: one from overflowing froth and another from beneath the froth which captures particles that fail to join the froth. Unlike other FBFCs like the HF, the NC is notable for its operation without desliming and achieves remarkable recovery rates for coarse particles^102^. This review found no application of the NC in spodumene beneficiation.

#### Reflux™

The RC through the application of a downward flow of wash water, creates a reverse fluidized bed. RCs can accommodate a wide range of particle sizes (+ 1 μm-2 mm) and ensures consistency in product grade. The RC integrates both a downcomer and a fluidized bed within a single apparatus. Key features incorporated below the main vertical section of the cell are inclined channels designed to accelerate the separation of bubbles from the liquid phase. The principle leveraged here is the Boycott effect, where the inclined channels facilitate enhanced multi-phase separation by promoting the upward movement of bubbles against the downward liquid flow^103^. Also, installed in RCs are downcomers, vertically aligned and on opposing sides along the elongated walls of the narrow channel to control flow dynamics. Sparger plates are installed to introduce air into the slurry flow under pressure. The high downward velocity of the feed slurry through the channel generates intense shear forces, facilitating the creation of fine bubbles and achieving exceptionally high bubble surface area fluxes. This unique configuration allows flotation of coarse and even ultrafine particles^104^. Dickinson et al.^105^ emphasized the capacity advantage of the Reflux™. They found that the Reflux cell significantly reduced the liquid flux overflow, and the inclined planes provided the capacity for shorter residence times under high shear rates, an advantage not achievable with only gravity. Reflux flotation machines, though not widely publicized, have been integrated into certain lithium processing plants.

**Table 5:** Summary and comparison of the main FBFCs.

Technology	Hydro float	Nova cell™	Reflux
Energy savings	(24–41)%^13^	(40–50%)^24^	20%^108^
Recovery rate	25% and 44% respective increase in recovery over the Denver cell for (425–850 μm and 850–1180 μm) for sphalerite particles^106^. 90% copper recovery for 500–800 μm^12^. Approximately 100% recovery of 850 − 500 *µ*m sulphide ore using both HydroFloat and conventional mechanical flotation cell^107^.	97% for 0–2 mm coal particles^24^.	82–97% recovery for + 0.125–2 mm coal tracer particles^102^
Operating principle	*downward fluidization* Uses air bubbles and fluidization bed to assist hydrophobic particles to report to the system overflow^109^.	*downward fluidization* Uses an aerated recycle stream to disperse air into very fine bubbles.	*upward fluidization* Utilizes reflux action with inclined channels to create a laminar flow regime within the cell.
Prominent features	*Dense phase fluidized bed*: Eliminates axial mixing, increases residence time for coarse particles^15^.	*Two tail streams*: The underflow transports the coarser dense high-solid content, while overflow carries the floated fine materials from the top of the column.	*Inclined parallel channels*: Enhance segregation between rising bubbles and descending liquid to prevent bubble entrainment^111^
Competitive advantages	Operates with little to no froth layer which is allowed to overflow. The hydraulic lift generated by the rising water current provides the necessary momentum for clusters to ascend and overflow, facilitating levitation of coarse particles.	Capable of delivering a major part of the tailings as a de-slimed slurry that can be dry stacked with minimum treatment^110^	Eliminates fine particle entrainment through a positive liquid bias flux, improving separation efficiency^24^ Able to float particles up to 2 mm, allowing potential replacement of gravity separation and classifying cyclone circuits with a single flotation circuit^102^.
Limitations	Difficulty in recovering hydrophobic particles over the classification size (+ 150 μm) . Feed typically requires desliming to prevent elutriation of fine gangue particles into the overflow stream^16^. The use of water as the fluidization medium could be problematic in arid areas^15^.	Presence of bubble-particle clusters beneath the froth layer may result in sub-optimal froth recoveries^24^	Operation demands precise control of liquid flux and feed conditions to maintain laminar flow. Existing research primarily focuses on coal feedstocks with low solids concentration.

## Ore sorting

Ore Sorting is a pre-concentration method used to separate mineral particles based on properties such as colour, density and electromagnetic susceptibilities. Sorting can be manual or automated. Manual sorting of spodumene relies on the visual distinctions in colour and shape between spodumene and associated gangue. Sorting is typically applied at particle sizes (10–25 mm)^112^. The earliest application of sorting in lithium operations was reported by Munson and Clarke^32^ at the Edison mine. This involved hand sorting of spodumene at the mine face, followed by the installation of a picking belt. The system consisted of a 12-inch rail grizzly, ore bin, 18 × 30-inch jaw crusher, a shaking screen with 1.5-inch square mesh cloth, transfer conveyor, surge bin and a 30-inch flat picking belt that was 30 feet long. The material below 1.5 inches was removed by a small lateral conveyor. The picking belt was positioned over the final ore bin, which was divided to collect both the reject material and spodumene concentrate. The concentrate was then hauled to the railroad for shipment, while the reject material was sent to waste dumps near the mine. About 0.4 tonne of barren pegmatite was rejected at the mine face before delivery to the sorting circuit.

However, difficulties with sorting of lithium minerals may arise from overlapping colours of the minerals. Brandt and Haus^113^ reported the use of optical sorting to separate spodumene from feldspar and quartz. This operation was complicated by the appearance of spodumene, which closely resembled its associated gangue impurities. Hence, technological advancements are leaning towards the adoption of machine-based sorting technologies like optical sorters and X-ray transmission sorters, which offer more efficient and accurate separation. The advantages of sensor-based ore sorting technology have been consistently demonstrated over the past decade. These systems facilitate rapid, high-throughput analysis of ore streams, enabling real-time identification and separation without the need for labour-intensive manual sorting. By precisely targeting high-grade ore particles, sensor-based sorting enhances recovery rates and maximizes overall yield, optimizing resource utilization^114^. It is important to note that insufficient mineral liberation can compromise the detection and ejection efficiency of ore sorting^115^.

Äijälä^116^ studied the spatial modelling of sorting properties specific to pegmatite ores. The study leveraged the contrasting colours of pegmatite ore and waste rocks to employ optical separation methods. Notably, the LASER sensor system exhibited high accuracy in identifying differences between spodumene pegmatite and barren pegmatite, accepted 88% of ore samples as pre-concentrate and rejected 12% as waste. In 2016, Galaxy Resources Australia sought to find a reliable solution for concentrating lithium minerals heavily contaminated with basalt. Upon exploring various options, successful testing identified the TOMRA PRO Secondary Laser sorter as a promising solution. Since its implementation in 2021, the sorter has effectively treated contaminated low-grade lithium ores at particle sizes 14–25 mm and 25–75 mm. The sorted product is then recirculated into the crushing circuit, crushed to a -14 mm particle size and directed for DMS. The target of this operation is to maintain the basalt content in the product below 4%. Remarkably, the sorter’s performance has remained consistent since its commissioning.

## Magnetic separation

Magnetic separation is an effective beneficiation technique commonly used to improve the purity of spodumene concentrates typically at size + 75–300 μm. This technique is essential when the concentrate needs to meet specific purity standards for different end uses. For instance, if a ceramic or glass-grade spodumene product requires a maximum allowable Fe_2_O_3_ content of less than 0.5%^8^. Magnetic separation techniques, such as high gradient magnetic separation (HGMS) and wet high-intensity magnetic separation (WHIMS), are commonly applied to spodumene processing. The sporadic presence of gangue minerals within the crystal lattice of spodumene complicates their removal through gravity and flotation methods. However, these gangue minerals are often magnetic, which makes magnetic separation effective for their removal. While magnetic separation is traditionally applied to the final spodumene concentrate, it is also viable for use during flotation to improve separation efficiency^9^.

Botula et al.^117^ demonstrated the successful recovery of zinnwaldite, from tin-tungsten mining tailings in the Czech Republic. The separation was conducted across a broad magnetic induction range (3500–7200 × 10⁻⁴ T). Similarly, Chehreh Chelgani et al.^118^ investigated the relationship between chemical analyses and magnetic susceptibility in zinnwaldite by magnetic separation of various size fractions. Their statistical analyses demonstrated that the magnetic susceptibility of zinnwaldite, could be accurately predicted based on the mineral’s cation content. The study also highlighted that particle size significantly influences magnetic susceptibility, with a small difference between the estimated and measured values for the non-linear relationship, which was less than 1 × 10⁻⁸ m³/kg. They suggested that such predictive techniques can be applied to estimate the magnetic properties of zinnwaldite in different resources even for in-situ analysis. Magnetic separators have been used to refine glass-grade spodumene concentrate from coarse fractions at the Greenbushes spodumene processing plant in Western Australia. WHIMS produced spodumene concentrate free of tourmaline within the non-magnetic fraction. The concentrate assayed (7.5%-7.7% Li_₂_O) with less than 0.1% Fe_2_O_3_^6^.

Furthermore, Bartkowska et al.^119^ conducted a study on the high-field magnetization and magnetic susceptibility of kunzite, a variety of spodumene mineral, in the temperature range of 1.6–400 K. Their findings revealed that the total magnetization and susceptibility of kunzite consist of both paramagnetic contributions from temperature-dependent Brillouin-type behaviour of magnetic ions and temperature-independent diamagnetic contributions from the spodumene matrix which was about equal to − 3.5 × 10 ^− 7^ emu/g. This study demonstrated the complex magnetic properties of spodumene and its varieties, further highlighting the challenges in separating spodumene from other minerals based solely on magnetic susceptibility.

## Economic and environmental benefits of coarse beneficiation

With increasing energy costs and declining ore grades, pre-concentration of low-grade hard rock lithium ores is receiving greater attention. To maximize energy efficiency, pre-concentration should be performed at the coarsest particle size possible with minimal value mineral loss^120^. Coarse particle beneficiation techniques can save up to 50% in energy consumption^13,115^. This is achievable with advanced circuits that reject coarse liberated gangue material early in the beneficiation process. By reducing the mass fed to comminution circuits, these methods not only improve throughput but also decrease energy consumption. Moreover, the rejection of waste before milling can improve feed grade to downstream concentrators, increase recovery and offset the loss of some valuable minerals during early-stage rejection. Furthermore, the early-stage rejection of silicate gangue can reduce slime and fine generation during grinding, thereby lowering reagent consumption (particularly collectors) during downstream flotation^121^.

A study conducted at SGS Lakefield in Manitoba involved metallurgical test work on a composite sample of pegmatite and waste rock from the Grass River Lithium Deposit, with a head sample assay of 1.24% Li_₂_O and 2.08% Fe_2_O_3_^122^. The program aimed to produce a concentrate with 6.0% Li_2_O and less than 1.0% Fe_2_O_3_. Ore sorting tests were carried out on the + 12.7 mm and + 9.5 mm fractions, which effectively rejected 20% of the feed mass as gangue with only a 3.2% lithium loss. The lithium grade increased from 1.43% Li_2_O to 1.72% Li_2_O in the ore-sorted product. This was followed by HLS tests at crush sizes of -6.3 mm and − 9.5 mm. At a concentrate grade of 6.0% Li_2_O, the lithium recovery from the − 6.3 + 0.85 mm fraction was 86.2% at 0.94% Fe_2_O_3_, which met concentrate specifications. For the − 9.5 + 0.85 mm fraction, the interpolated lithium recovery was 79.2% at 6.0% Li_2_O and 1.18% Fe_2_O_3_. After DMS, the concentrate was passed through dry magnetic separators, which produced a non-magnetic DMS concentrate grade assaying 6.5% Li_2_O and 0.98% Fe_2_O_3_ at 67.5% recovery^122^.

Studies by Legault-Seguin et al.^120^ showed that mass rejection rates of 20–60% can be achieved while maintaining lithium recoveries over 90%. For example, at the Nemaska Lithium Whabouchi Project, DMS flowsheet development aimed to reduce downstream flotation requirements. Pilot-scale tests involved crushing, scrubbing, screening, DMS and magnetic separation, to produce a concentrate which was 13% of the feed mass at 6.0% Li_2_O with approximately 50% lithium distribution. The middlings fraction, which was 45% of the feed mass, averaged 1.6% Li_2_O, while lithium losses to tailings remained below 10%, with over 40% mass rejection. Awatey et al.^13^ compared the metallurgical performance and energy consumption of two flowsheets: one incorporating the HydroFloat separator in a conventional flotation setup (Flowsheet 1) and the other using a single-stage Denver mechanically agitated flotation cell (Flowsheet 2). Flowsheet 1 achieved a 23% reduction in energy consumption compared to Flowsheet 2, with the added benefit of reducing primary grinding media consumption, which further enhanced its economic appeal.

Reduced grinding directly translates to a decrease in greenhouse gas emissions due to the reduction in fossil fuel energy consumption^115^. This can contribute to a lower carbon footprint in lithium operations. Furthermore, coarse particles with their lower specific surface area, require significantly lower reagent dosages during flotation conditioning compared to finer particles. Reduced chemical consumption can reduce processing costs and risk of exposure to harmful chemicals. FBFCs can also be used to process coarse tailings and reduce the leaching of harmful substances into the environment. This can lead to more sustainable waste practices, as the reduced fines in tailings can mitigate risks of soil and water contamination. Coase particle processing supports environmental stewardship by improving waste management which aligns with sustainability goals in the lithium mining industry.

## Conclusions and future perspectives

Coarse particle beneficiation methods applicable to lithium minerals have been discussed, with a primary focus on spodumene, as it is currently the most commercialized lithium-bearing mineral in pegmatites. Future research should consider other lithium-bearing minerals such as petalite, lepidolite and zinnwaldite. At the coarse particle level, gravity concentration using DMS is the most effective technique. With adequate liberation at coarse particle sizes, DMS can effectively preconcentrate spodumene and reject gangue minerals such as tantalite and cassiterite. Future studies should explore the use of EGS such as Knelson concentrators, Falcon separators and multi-gravity separators which have potential applications to improve the recovery of both coarse and fine particles.

Fine particle flotation using mechanical flotation cells has been combined with DMS to effectively recover fine particles. However, as comminution costs rise, process economics could be optimized by focusing on the recovery of coarse particles that might otherwise be lost in conventional flotation cells. FBFCs such as HF, NC and RC can offer alternative approaches for coarse particle flotation of lithium-bearing minerals by reducing the need for fine grinding, lowering energy costs and enhancing recovery rates^2^. Among these, the HydroFloat is the most effective for spodumene flotation, as it combines density separation with flotation selectivity. While the NovaCell Reflux typically excel across fine to coarse particle recovery, the HydroFloat can offer the best balance for coarse spodumene flotation. Despite the advantages of FBFCs, their applications in lithium operations are very few and limited to pilot scale, with several challenges that must be addressed before commercial-scale up. Also, the relationships between various operational parameters of these technologies require further exploration^95^. One significant challenge is the high-water demand for fluidization in FBFCs, which could be alleviated by integrating water recycling units. Additionally, pre-classification issues particularly in the HydroFloat can be mitigated by optimizing grinding techniques to minimize the generation of fines.

Pretreatment methods such as alkali treatment, calcination, ultrasonic treatment, and isomorphous substitution have shown significant potential for improving selectivity in spodumene beneficiation^50,73,74^. Among these, grinding additives are the most widely used due to their economic feasibility for large-scale operations. NaOH pretreatment has been found to enhance spodumene flotation, making it suitable for direct flotation, while HCl or Na₂CO₃ pretreatment can be beneficial for reverse flotation. The addition of multivalent metal ions (Ca^2+^, Cu^2+^, Mg^2+^, Fe^2+^, Al^2+^) has been shown to improve flotation efficiency by forming stable metal-hydroxyl complexes with anionic collectors. Studies by Xie et al.^51^ and Meng et al.^78^ have demonstrated that Mg^2+^ and Ca^2+^ are the most effective ions for spodumene activation. Further research into the mechanisms behind the adsorption of these metal ions and collectors would be beneficial, as the interactions are not fully understood. Also, the development of new collectors with superior selectivity and collectability for spodumene flotation is promising^57^. Mixed collector systems, which combine the synergistic benefits of cationic and anionic collectors, have demonstrated enhanced flotation efficiency due to their improved selectivity and adsorption capability on spodumene surfaces. However, further research is required to enhance the applicability of mixed collectors at industrial scale.

The integration of sensor-based ore sorting technologies, such as the TOMRA PRO sorter, allows for high-throughput, real-time analysis to precisely separate lithium-bearing minerals from waste. The effectiveness of sensor-based sorting can be enhanced through improved mineral liberation. Future advancements could focus on developing advanced sensors which rely on X-ray transmission (XRT) and laser-induced breakdown spectroscopy (LIBS), to improve the identification and separation of complex ore matrices. Spodumene exhibits weak paramagnetism due to the presence of iron impurities such as hematite. In contrast, diamagnetic minerals such as feldspar, quartz and mica remain unaffected by magnetic fields. This enables the selective separation of spodumene from non-magnetic gangue minerals. Although magnetic separation cannot function as a standalone beneficiation method, its application during or after flotation can improve the purity of spodumene concentrates.

## Data Availability

The datasets used and/or analysed during the current study available from the corresponding author on reasonable request.
